# Mexican and Puerto Rican Men’s Preferences Regarding a Healthy Eating, Physical Activity and Body Image Intervention

**DOI:** 10.3390/nu14214634

**Published:** 2022-11-03

**Authors:** Lisa Sanchez-Johnsen, Amanda Dykema-Engblade, Carlos E. Rosas, Leonilda Calderon, Alfred Rademaker, Magdalena Nava, Chandra Hassan

**Affiliations:** 1 Department of Family and Preventive Medicine, Rush University Medical Center, 1645 West Jackson Blvd, Suite 302, Chicago, IL 60612, USA; 2 Department of Psychology, University of Illinois at Chicago, 1007 W Harrison St., Chicago, IL 60607, USA; 3 Department of Psychology, Northeastern Illinois University, 5500 North St. Louis Ave, Chicago, IL 60625, USA; 4 Puerto Rican Cultural Center, 2628 W. Division St., Chicago, IL 60612, USA; 5 Department of Preventive Medicine, Feinberg School of Medicine, Northwestern University, 680 N Lake Shore Drive, Suite #1400, Chicago, IL 60611, USA; 6 Department of Obstetrics and Gynecology, Feinberg School of Medicine, Northwestern University, 625 N. Michigan Ave Suite #1100, Chicago, IL 60601, USA; 7 Department of Surgery, University of Illinois at Chicago, 840 South Wood Street (MC 958), Chicago, IL 60612, USA

**Keywords:** Latino men, obesity, intervention, diet, body image, physical activity

## Abstract

This study examined the logistical, practical, and cultural preferences of Latinos regarding the design of a healthy eating, physical activity, and body image intervention. Puerto Rican and Mexican men (*n* = 203) completed an interview as part of an NIH-funded study. Overall, 66.5% preferred the intervention to be in Spanish only or both Spanish and English; 88.67% said it was moderately, very or extremely important for the intervention leader to be bilingual; and 66.01% considered it moderately to extremely important for the leader to be Hispanic or Latino. Most participants (83.74%) reported they would be willing to attend an intervention that met twice per week and 74.38% said they would be willing to attend an intervention that met for 1.5 to 2 h, twice weekly. Overall, the majority said they would be moderately to extremely interested in attending an exercise program if it consisted of aerobics with Latin or salsa movements (74.88%) and if it consisted of aerobics with Latin or salsa music (70.44%). Some participants were moderately to extremely interested in attending an intervention if it included *dichos* (Latino sayings) (65.02%) and *cuentos* (folktales or stories) (69.46%). The findings have implications for lifestyle and body image interventions aimed at preventing cardiometabolic diseases.

## 1. Introduction

Among men, overweight (BMI ≥ 25) and obesity (BMI ≥ 30) are serious health issues that are associated with numerous health problems, including cardiometabolic diseases [1,2,3,4]. Latino men, in particular, have concerningly high rates of overweight or obesity. In fact, Hispanic/Latino men have the highest prevalence (41.2%) of obesity or severe obesity compared to non-Hispanic Black (37.5%), non-Hispanic White (36.9%), and Asian (11.7%) men [5]. Moreover, there are marked differences in overweight and obesity rates between Latino men of different backgrounds. In the *Hispanic Community Health Study/Study of Latinos* (HCHS/SOL), a landmark epidemiologic study of Hispanics/Latinos, men of Puerto Rican (40.9%) and Mexican (36.8%) descent—the two largest Hispanic/Latino subgroups in the U.S. [6]—had the highest rates compared to men of other Hispanic/Latino backgrounds [7]. The differences in the prevalence of overweight and obesity between Hispanics/Latinos and other racial/ethnic groups and between different Hispanic/Latino ethnic groups are attributable to behavioral and environmental factors in addition to genetic influences [8,9]. Given the influential role of obesity in development of cardiometabolic diseases [3], addressing these disparities is critical. Behavioral weight loss interventions are the preferred method for treating overweight and obesity; however, few weight loss interventions have been developed specifically for Latino men [10,11] and fewer, if any, have focused on specific subgroups of Latinxs.

The majority of diet and physical activity interventions conducted with Hispanic/Latinos have primarily targeted women, e.g., [12,13,14,15,16]; and only a few, to our knowledge, have focused exclusively on Hispanic/Latino men [17,18]. Indeed, the dearth of physical activity interventions for Hispanic/Latino men has been noted in two recent systematic reviews [10,11]. The low number of Hispanic/Latino men in weight-loss interventions is surprising given that studies indicate that, in general, men are interested in interventions that address overweight and obesity [19].

Despite this interest, men tend to enroll less frequently in behavioral weight loss interventions than women [20], and this disparity is exacerbated among Hispanic/Latino men [21,22]. Lack of enrollment of Latino men in weight loss interventions is two-sided. On one hand, Latino men face logistical (e.g., limited off-work time) and sociocultural (e.g., lack of control of diet due to gender roles) barriers that make it difficult to adopt healthy lifestyle behaviors [21,22]. For example, in a study among 14 Latino men with overweight, Garcia et al. [21] found that some of the barriers to healthy eating and physical activity included dietary norms and familial influence, long work hours, and fatigue. On the other hand, there is a lack of interventions that are designed to address the specific preferences and needs of Latino men [23]. While some reasons for the lack of enrollment—such as limited off-work time—among men are similar across racial/ethnic groups, others are specific to Hispanic/Latino men (e.g., spoken language, living environment, social, cultural, and familial influences) [21,23]. Factors that may be important to address these needs and overcome some barriers include the language of the intervention and program materials, the characteristics of the intervention leader, and the location and type of physical activity [21,24], and the integration of cultural elements into the intervention.

Moreover, interventions that focus on Latino men are important due to their patterns of diet and physical activity. For example, in the HCHS/SOL, individuals of Puerto Rican descent had the lowest estimated intake of fruits and vegetables compared to other Hispanic/Latino groups [25]. Further, a large proportion of Hispanic/Latino men (>73%) do not engage in regular physical activity [26]. Data from a recent study revealed that number of hours watching television per day was higher among Puerto Rican than Mexican men [27]. In another study, 47.5% of Mexican men and 56.9% of Puerto Rican men reported no leisure-time physical activity [28].

According to recent U.S. Census data, 67.6% of Hispanics/Latinos speak Spanish at home, and of those, 22.8% do not speak English well [29]. Language preference, however, varies by nativity and Hispanic/Latino background, with U.S.-born and Puerto Ricans more likely to speak English only than foreign-born and other Hispanics/Latinos [30]. Thus, an English- or Spanish-only intervention may not be appropriate for all Hispanic/Latino groups. Moreover, some Latinx groups (e.g., less acculturated groups) may prefer a Latinx intervention leader whereas others may not have a specific preference for the ethnicity of the leader. In a past qualitative study, a sample of 39 Mexican American women and men expressed that the ideal physical intervention would be led by a bilingual instructor [31]. However, no study, to our knowledge, has examined the preferences regarding the ethnicity of intervention leaders among Latinx men.

Another important consideration for interventions is the location of the intervention. Indeed, inadequate access to or lack of safe spaces for physical activity has been identified as a major barrier among Hispanic men [18,23,31]. Hispanic men report that their neighborhoods may be unsafe (e.g., gang activity) or inadequate (e.g., lack sidewalks and/or streetlights) for exercise, but, at the same time, transportation to and from facilities outside of their communities may be time-consuming [18,23,31]. While past studies have assessed Latinx men’s perceived barriers, few, if any, have inquired about their preferences. Relatedly, preferences for specific types of physical activity reported in previous studies are mixed, with some Latino men preferring team sports [18,32] and others walking [31]. Despite that dancing-based aerobic exercises are easier to implement (e.g., can be performed in- or out-doors, requires less space) than other forms of physical activity and are popular among Latinx men [33], little is known about Latinx men’s preferences regarding aerobics-based interventions.

Culture-specific content are also important to include in interventions that address diet and physical activity among culturally and linguistically diverse populations [34]. One type of cultural-specific content is *dichos*, or Latino sayings or proverbs. Although *dichos* have not been used in a combined diet, physical activity and body image intervention for Latino men, they have been used in counseling [35,36], educational or health literacy programs [37,38], and in other interventions among Latinos [39,40,41]. For instance, *dichos* have previously been used in the development of an intervention focused on nutrition for Latinas and their families [42], and in another one focused on diet, physical activity, body image, and secondhand smoke intervention with Latina women [43]. Specifically, *dichos* can be validating to a person, can communicate openness to a person’s Latino culture, and can be used to build rapport and trust [35]. Thus, *dichos* may be a culturally appropriate strategy to recruit, improve communication, and encourage healthy lifestyle behaviors among Latinos [38]. It is unknown, however, whether including *dichos* in an intervention would make it more appealing for Latino men. Similarly, the use of *cuentos*—folktales or stories/storytelling in Spanish—has yet to be examined in a health intervention with Latino men. Although not used extensively, *cuentos* have also been used in therapy and interventions to promote personal and social development and healthy behaviors [44,45,46]. If *dichos* and *cuentos* are important for Latino men, then such cultural components may be used to guide the development of a diet, physical activity and body image intervention for Latino men.

Latino men have been largely absent from interventions seeking to promote healthy lifestyles, and it is possible that this omission is due to the lack of tailoring interventions to their specific needs and preferences. Moreover, few studies, if any, have assessed differences in needs and preferences among different Latinx groups. To this end, the present study aimed to examine the preferences of Mexican and Puerto Rican men regarding the design and development of a healthy eating, physical activity and body image intervention. Specifically, the following were examined: Preferences regarding the language of the intervention and intervention materials, the importance of the intervention leader’s language and Hispanic or Latino background; preferences regarding the frequency, duration, and location of the intervention; preferences regarding the type of exercise program; and questions regarding the use of various cultural elements such as *dichos* and *cuentos* during the intervention.

## 2. Methods

### 2.1. Sample

Data for the present study comes from the Latino Men’s Health Initiative, which was an NIH-funded cross-sectional study (R21CA143636) designed to explore the role of cultural variables underlying race and ethnicity (acculturation, acculturative stress, ethnic identity, and cultural values) as they related to diet, physical activity, and body image among normal weight, overweight and obese Mexican and Puerto Rican men [47]. The Latino Men’s Health Initiative relied on the heavy involvement of community partners who work with Latinos for a complete description, see [47]. Relying on community partners’ experiences and expertise is consistent with the community-based participatory research (CBPR) model and allows for community partners to be involved in all aspects of the research process (e.g., recruitment, designing of interventions, dissemination of results) [48]. Data collection for the overall parent study (Latino Men’s Health Initiative) was funded between 2010 and 2014. Overall, results from the Latino Men’s Health Initiative will be used to gain insight into the best ways to develop diet, physical activity and body image interventions for Mexican and Puerto Rican men using community-engaged and culturally targeted approaches.

Two hundred three Latino men (99 Mexicans and 104 Puerto Ricans) participated in the study. As noted elsewhere [47], the sample consisted of the following weight categories: Mexicans: 31.31% (*n* = 31) normal weight (BMI = 18.5–24.99), 36.36% (*n* = 36) overweight (BMI = 25–29.99), and 32.32% (*n* = 32), obese (BMI > 30); Puerto Ricans: 35.58% (*n* = 37) normal weight, 32.69% (*n* = 34) overweight, and 31.73% (*n* = 33) obese.

Inclusion criteria were as follows: (a) Self-identified Mexican or Puerto Rican men, even if they identified as biracial. (b) Ages 18–65. (c) Those who agreed to provide informed consent. Exclusion criteria were as follows: (a) Men with a BMI lower than 18.5 kg/m^2^ (no upper BMI limit). (b) Men who were unable to speak/read English or Spanish. (c) Those with an eating disorder (Bulimia Nervosa, Anorexia Nervosa, Binge Eating Disorder). (d) Men who had the intention to leave the Chicagoland area over the course of the study (i.e., 6 weeks).

### 2.2. Recruitment

The current study utilized both direct and indirect forms of recruitment. Direct recruitment involved distributing flyers at Hispanic/Latino community organizations, churches, festivals, and health fairs. The flyers promoted a free culture and health research study for Latino males. Additionally, flyers were provided to health care professionals, community members and posted in various locations throughout Chicago. Indirect recruitment consisted of newspaper advertisements, newsletters, letters to doctors and other organizations focused on Latino and/or health issues, postings to email listserves and websites, and at the University of Illinois at Chicago. For a thorough description of the recruitment strategies, see Sánchez-Johnsen et al. [47].

### 2.3. Study Procedures

Oral consent was obtained prior to determining an individual’s initial eligibility. Once oral consent was obtained, research assistants proceeded to administer a 30 min eligibility interview via telephone or in-person. For those who met the initial eligibility criteria, a face-to-face visit was scheduled to complete the written consent. After written informed consent was obtained in-person, height and weight were measured using a stadiometer and Seca company digital scale, respectively. Body Mass Index [(BMI) weight (kg)/height (m)^2^)] [49] was calculated to determine eligibility. As part of the larger NIH-funded study (R21CA143636), eligible participants completed a two and a half hour “Health and Culture” interview, which assessed their demographic and background characteristics, health and diet choices, weight, culture (e.g., cultural values and beliefs), exercise patterns, body image, and social support questions, and preferences about the development of a future intervention focused on healthy eating, physical activity, and body image See [47] for additional details. To assess participants’ preferences regarding the development of a future intervention, they were given the following instructions: “At some point in the future, we will be developing a healthy eating, physical activity, and body image program that will focus on Latinos. In the next part of the interview, we will ask about your opinions and preferences concerning the development of a healthy eating, physical activity, and body image intervention in the future. Your responses will help us to identify certain things that may need to be considered when developing these programs in the future”. Twenty percent of the overweight and obese sample were also asked to complete an in-depth qualitative interview adapted from [50]. Participants were also asked to complete a diet questionnaire and to use an accelerometer for seven days and record the results. The following additional objective measures were obtained: Bioelectrical Impedance Analysis, using a Tania BF 682 [51] to assess body fat, and waist and hip circumference, to assess fat distribution.

Participants were compensated for completing the health and culture interview ($50) and the other components of the study ($10 for completing the diet questionnaire, $15 for using the accelerometer and recording the results, and $40 for completing the qualitative interview). The Institutional Review Board at the University of Illinois at Chicago (IRB 2011-0187) and the Research Review Board at Alivio Medical Center approved this study.

### 2.4. Community Partners

In the best effort to meet the needs of community members, the first author collaborated with community partners who served as members of the Hispanic/Latino Health Community Advisory Board (HLH-CAB). Prior to recruitment, HLH-CAB members assisted with the development of the recruitment plan and study methods, as well as provided critical insights and feedback about the content and design of the questionnaires, interview materials, informed consent document, incentives, and objective measures details can be found in [47]. Overall, along with the study team, the HLH-CAB sought to conduct the study at the highest level of cultural proficiency and to ensure that the study was relevant to the Mexican and Puerto Rican communities, while paying attention to differences within and among Latino communities.

### 2.5. Measures

#### 2.5.1. Translation of Measures

All study materials were available in English and Spanish. Measures not available in Spanish were translated as recommended by numerous authors, e.g., [52,53]. Translated materials were then reviewed by members of the HLH-CAB and bilingual research assistants through a community engaged research process See [54] for additional information about the translation process.

#### 2.5.2. Demographics

Demographic information, including information about their specific Latino background, race, age, marital status, education, occupation, and nativity, was self-reported. Descriptor variables included in the study were questions assessing demographics, literacy level, socio-economic status [55,56], and various additional questions assessing inclusion and exclusion criteria, e.g., [57]

#### 2.5.3. Intervention Language and Ethnicity of Intervention Leader

Questions were posed about the design of a future intervention focused on healthy eating, physical activity and body image. Participants were asked about their language preferences regarding an intervention [language of the intervention and language of the intervention materials (e.g., handouts, instructions), respectively]. Response choices included: (1) Spanish only, (2) English only, (3) Both Spanish and English or (4) Other. Participants were also asked questions about the importance of the intervention leader to be Hispanic or Latino and the importance of the leader to be bilingual. Response choices were (1) not at all important, (2) not very important, (3) moderately important, (4) very important, or (5) extremely important.

#### 2.5.4. Structure of Intervention

Questions were also asked about the frequency and duration of the intervention. Specifically, participants were asked, “Would you be willing to attend an intervention that met twice per week?” and “Would you be willing to attend an intervention that lasted for about one and a half to two hours on the first day and one and a half to two hours on the second day? Response choices for each question were “yes” or “no”. Participants were also asked to rate their interest in attending an intervention if it was located in certain areas. Three of the four areas included in the questionnaire are located in Chicago neighborhoods that are traditionally considered as comprising large Mexican (Pilsen, Little Village) and/or Puerto Rican (Humboldt Park) communities. In addition, participants were asked how interested they would be to attend an intervention at University of Illinois at Chicago (the university where the study PI was a faculty member at that time). Interest was rated on a 5-point Likert scale, which ranged from (1) Not at all interested to (5) Extremely interested.

#### 2.5.5. Exercise Program

Preferences regarding the type of exercise program was also assessed. Participants were asked how interested they would be to attend an exercise program if the exercise consisted of aerobics with Latin or salsa music, or if the exercise consisted of aerobics conducted with Latin or salsa movements. Response choices ranged from (1) not at all interested to (5) extremely interested on a 5-point Likert scale.

#### 2.5.6. Integration of Cultural Elements

Finally, questions were asked about the inclusion of cultural elements called *dichos* (Latino sayings or proverbs) and *cuentos* (stories or storytelling). Participants were first asked whether they have heard of *dichos*. If they responded yes, then they were asked if they have heard of *dichos* related to exercise, physical activity, or dancing; and whether they have heard of *dichos* related to their body or to health, respectively. They responded either “yes” or “no”. Participants were then asked how interested they would be in participating in an intervention if it included *dichos*, in order to make it more culturally meaningful for Latinos. Response choices ranged from (1) Not at all interested to (5) Extremely interested on a 5-point Likert scale.

Similarly, participants were first asked whether they had heard any *cuentos* (stories or story-telling). If they responded affirmatively, they were then asked whether any of those cuentos related to exercise, physical activity, or dancing; and whether they have heard of *cuentos* related to their body or to health, respectively. After those questions, participants were asked how interested they would be in participating in an intervention that included *cuentos,* in order to make it more meaningful for Latinos. Participants were asked to respond to these questions using a 5-point Likert scale, which ranged from (1) not at all interested to (5) extremely interested.

### 2.6. Statistical Analyses

Frequencies and percentages were computed for sociodemographic factors. Responses were compared between the Mexican and Puerto Rican groups using the chi-square test of independence. Data were first analyzed using the overall sample of all weight groups. Given our interest in designing an intervention for individuals with overweight or obesity, a second analysis compared the groups restricting the analyses to those who were overweight and obese (BMI ≥ 25 kg/m^2^). SPSS was used for all analyses and statistical significance was indicated if *p* < 0.05.

## 3. Results

### 3.1. Sample Characteristics

Demographic and background characteristics have been reported elsewhere [47] and summarized here. Four-hundred and thirty-five participants contacted members of the research team, 344 completed the oral script and initial eligibility interview, 211 men completed the written consent, 203 men completed the “Health and Culture” interview, and 193 completed all study components which included the “Health and Culture” interview, the diet questionnaire, and the use of an accelerometer. The data reported here are from the 203 Latino men (99 Mexican and 104 Puerto Ricans) who completed the “Health and Culture” interview. Participants’ ages ranged from 18 to 65 years (*M* = 39.4, *SD* = 12.3). Most were single/never married (52.7%; *n* = 107) and had a high school diploma or equivalent (GED) or higher or some college (1–3 years) or graduated from a 4 year college (66.0%, *n* = 134). As intended, participants were roughly equally distributed between the three weight categories: 31.5% Normal, 34.5% Overweight, and 32.0% Obese.

There were significant differences in the following variables: age, with Puerto Rican being older (*p* < 0.0001), less likely to be single/never married (*p* < 0.05), more likely to have been born in the U.S. mainland (*p* < 0.05), more likely to smoke (*p* < 0.05), and more likely to be unemployed (*p* < 0.001) than Mexicans. Other characteristics including the highest grade completed, the percent of men who had health insurance, religion and the language in which the interview was completed showed no significant differences between Puerto Ricans and Mexicans. Finally, there were no significant differences between Puerto Ricans and Mexicans in weight-related measures (i.e., BMI, body fat, hip and waist measurements).

### 3.2. Intervention Language and Ethnicity of Intervention Leader

As seen in Table 1, results revealed that 66.50% reported the language which they preferred the intervention to be conducted was Spanish or Spanish and English and 69.46% reported that the preferred language of the intervention materials was in both Spanish and English. Results revealed that 88.67% said it was “moderately”, “very” or “extremely important” for the intervention leader to be bilingual and 66.01% said it was either “moderately”, “very” or “extremely important” for the intervention leader to be Latino.

### 3.3. Structure of Intervention

In terms of the frequency and length of the intervention, results revealed that 83.74% said they would be willing to attend an intervention that met twice per week, and 74.38% said they would be willing to attend an intervention that met for one and a half to two hours on the first day and one and a half to two hours on the second day. There were no significant differences between Mexican and Puerto Rican men in terms of these responses. Results were similar in terms of level of responses and differences between ethnic groups when analyses were restricted to overweight and obese participants only (Table 1).

As seen in Table 2, participants reported they would be “moderately” “very” or “extremely interested” to attend an intervention if it were in the following Chicago neighborhoods: Humboldt Park (75.37%), the University of Illinois at Chicago area (72.41%), Pilsen (48.28%), and Little Village (38.92%). More Puerto Ricans than Mexicans preferred the location to be in Humboldt Park (*p* < 0.0001); more Mexicans than Puerto Ricans preferred the University of Illinois at Chicago (*p* = 0.009), Pilsen (*p* < 0.0001) or Little Village (*p* = 0.003). These preferences were similar when the sample was restricted to overweight and obese participants only (Table 2).

### 3.4. Exercise Program

As also seen in Table 2, 74.88% of men said they would be “moderately”, “very” or “extremely interested” in attending an exercise program if it consisted of aerobics with Latin or salsa movements, with Puerto Ricans indicating a greater preference (*p* = 0.047). Overall, 70.44% of men said they would be “moderately” “very” or “extremely interested” in attending an exercise program if the program consisted of aerobics with Latin or salsa music with no difference between ethnic groups. Among the overweight and obese sample, Puerto Ricans indicated a greater preference for both Latin or salsa movements (*p* = 0.011) or music (*p* = 0.042) (Table 2).

### 3.5. Integration of Cultural Elements

As seen in Table 3, 80.30% reported that they heard of *dichos*. Moreover, 59.51% reported that they heard of *dichos* related to food or eating, 46.63% reported that they heard of *dichos* related to exercise, physical activity or dancing, 54.60% reported that they heard of *dichos* related to their body, and 56.44% reported that they heard of *dichos* related to health. Overall, 65.02% reported that they were moderately, very or extremely interested in attending an intervention if it included *dichos*. In the overall sample, 69.46% reported that they were moderately, very or extremely interested in attending an intervention if it included *cuentos*, with the Mexican sample indicating a greater preference (*p* = 0.012). Results were similar in terms of level of response and ethnic differences when the sample was restricted to overweight and obese participants only (Table 3). In addition, in the overweight/obese sample, more Puerto Ricans than Mexicans reported that they heard of *dichos* related to exercise, physical activity or dancing (*p* = 0.04).

## 4. Discussion

Results from this study offer important insights about how interventions that address healthy eating/diet, physical activity and body image may be tailored to Mexican and Puerto Rican men. Results revealed that over half of the participants (66.5%) preferred the intervention to be in Spanish or Spanish and English. In addition, 88.68% of participants indicated that it was “moderately”, “very” or “extremely important” for the intervention leader to be bilingual and 66.01% said it was either “moderately” to “extremely important” for the intervention leader to be Latino. These findings highlight the importance of bilingualism among Mexican and Puerto Rican men. It is particularly important to note that although only two-thirds of participants preferred interventions to be in Spanish or Spanish and English and for the leader to be Latino, almost all participants indicated they would prefer a bilingual leader. Our findings replicate a previous study among Mexican Americans that identified having a bilingual intervention leader/instructor as a key component of the ideal physical activity intervention [31]. This suggests that, in interventions among Puerto Rican or Mexican men, having a bilingual leader is of paramount importance.

A high percentage of respondents indicated that 1.5–2 h meetings twice a week would be feasible. Previous qualitative research has found that some of the most significant barriers to physical activity among Latino men are busy work schedules and fatigue likely due to strenuous jobs [21,23,32]. Tailoring interventions to fit within Latino men’s busy schedules can help with recruitment and retention. Our findings suggest that meeting for up to two hours twice a week to engage in physical activity may be an appropriate starting point for community interventions. Although we did not specifically assess preferences for a virtual or telehealth intervention, future interventions may wish to also consider this option, while meeting twice weekly.

In addition to time, location of intervention is critical. In this study, Puerto Rican and Mexican participants reported a preference for different Chicago neighborhoods. Understandably, the results indicated that intervention location preferences follow the general ethnic composition of the same neighborhoods. That is, Puerto Ricans preferred interventions to be held in Puerto Rican neighborhoods and Mexicans in Mexican neighborhoods. Participants may feel more comfortable participating in interventions held in their communities. Alternatively, it is possible that participants chose a location based on the distance between the location and where they live, work, or how close that site is located to other places they visit, regardless of the ethnic composition of the community. Distance travelled to an intervention would be crucial in maintaining participant intervention adherence to the intervention program. Moreover, intervention location may have implications for long-term sustainability as participants may continue engaging in physical activity at the location of the intervention. Indeed, lack of access to safe spaces for exercise has been identified as a barrier to physical activity among Latinos [21,31].

In terms of the type of exercise program, the Puerto Rican participants with overweight/obesity indicated a significantly greater interest in exercise programs with Latin or salsa movements and music. This finding underscores the often-overlooked diversity within the Latinx community. In this study, Puerto Ricans showed a greater preference towards exercise programs with salsa movements and music than Mexicans. Usually, salsa music is more associated with Puerto Ricans or Puerto Rican music [58]. However, musical preferences among Latinxs vary; thus, it may be important for aerobics-based physical activity interventions to include culturally appropriate music for the target Latinx subgroup [11]. It should be noted, however, that the present study focused on the preferences of Latino men regarding an exercise program which included aerobics. While interventions that have included aerobics or dancing have been successful [59,60], other types of activities should be considered. Walking, for example, seems to be the preferred type of physical activity among individuals of Mexican ancestry [31,61].

Slightly more than half of all participants reported that they would be interested in attending an intervention that included *dichos* or *cuentos*. These findings suggest that *dichos* and *cuentos* may still be an important tool to include in interventions, albeit not as significant as other aspects (e.g., language of leader). Alternatively, the lower-than-expected interest in *dichos* and *cuentos* may be due to the age distribution and/or generational status of the participants. We will explore these factors in future analyses. Despite the widespread use of *dichos* and *cuentos* among Latinxs, there is a dearth of studies examining their role on physical activity and diet interventions in this group. Miranda et al. [38] suggest that *dichos* may serve as mantras of motivation for adopting healthy behaviors; however, they cautioned that *dichos* may not result in pragmatic changes for all. *Cuentos* may serve a similar role. Additional research, however, is warranted to examine the role and effectiveness of both *dichos* and *cuentos* in lifestyle and body image interventions.

This study has a number of limitations. First, it must be kept in mind that the current study is exploratory, and, therefore, interpretation of the results should be made cautiously. Second, the participants of this study were recruited from neighborhoods in Chicago, IL, thus the representativeness of the sample is limited. Third, the acculturation level, ethnic identity, and other background characteristics, such as place of residence, nativity, years in the U.S., and marital status, were not examined in relation to these findings or controlled statistically. This is an important area of future investigation and future studies will seek to further explore these variables as it relates to intervention preferences of Latino men. Fourth, questions that were reported in this paper were posed in a closed-ended fashion. Our future work will explore the physical activity responses of Latino men using an open-ended format, which will add to a deeper understanding of the preferences of Latino men. Finally, although it is a strength that this study included data on Mexicans and Puerto Ricans—two of the largest Latinx groups in the U.S.—there is a tremendous amount of diversity within and between these and other Latinx groups (e.g., acculturation) that should also be kept in mind when developing interventions for Latino men.

These limitations notwithstanding, a number of strengths must be highlighted. First, this study focused exclusively on Mexican and Puerto Rican men, a greatly understudied group, with a high prevalence of overweight and obesity. Second, a rigorous community-based protocol for recruitment and data collection resulted in invaluable information on design and delivery strategies for gender- and culture-sensitive interventions. Finally, together with results from our prior studies, the current study adds important insights that may help with designing healthy eating/diet, physical activity, body image, and overall overweight and obesity interventions for Mexican and Puerto Rican men.

Despite the need to involve Latino men in diet and physical activity interventions, more is needed to increase recruitment, adherence, and engagement in this group. One size fits all interventions have not been successful, in part, because Latino men have their own gender- and ethnicity-related set of preferences, barriers, and motivators in regard to diet and physical activity. First, in terms of language preference, future interventions should consider including bilingual intervention leaders and conducting the intervention itself in both languages (Spanish and English). Second, in terms of the structure, the Mexican and Puerto Rican men in this study preferred the intervention to be held twice per week for one and a half to two hours. This may provide a starting point for developing healthy eating, physical activity, and body image interventions. This study also illustrated the importance of selecting intervention locations, exercise programs (aerobics with Latin or salsa movements and music), and cultural elements (use of *dichos* and *cuentos*) that matched the preferences of men in this study. Such considerations should be taken into account when designing and delivering a healthy eating, physical activity, and body image intervention among Mexican and Puerto Rican men. In conclusion, our study provides critical information about the intervention preferences of Mexican and Puerto Rican men that may be used to guide the design and development of Latinx-centered obesity interventions for men in an effort to reduce risk of cardiometabolic diseases.

## Authors’ Notes

Parts of this paper were presented as an oral presentation at the 8th Annual University of Illinois at Chicago Minority Health Conference, Chicago, Illinois, April 2017, and at the Fourth International Culturally Responsive Evaluation and Assessment Conference, Chicago, Illinois, September 2017. Parts of this paper were also presented as a poster at the Latino Health Symposium, Medical Organization for Latino Advancement (MOLA), Chicago, Illinois, November 2017, where it also received the Best Poster Award for a Research Project. This work was conducted when Magdalena Nava was at the Puerto Rican Cultural Center.

The words ‘Hispanic’, ‘Hispanic/Latino’, and ‘Latinx’ are used interchangeably, depending on the study being reviewed. ‘Latinx’ is used as a general inclusive term to refer to all persons of Hispanic/Latin American background. The term ‘Latino’ is used for participants in the current study, as that was the term used in recruitment and/or interviews in the parent study (the Latino Men’s Health Initiative).

## Figures and Tables

**Table 1:** Preferences for Language, Hispanic/Latino Background, Length, and Frequency of the Intervention among All Participants and among Participants with Overweight or Obesity.

Preferences: *n* (%)	Overall (*n* = 203)	All Puerto Ricans (*n* = 104)	All Mexicans (*n* = 99)	*p*	PR with Overweight/ Obesity (*n* = 67)	MX with Overweight/ Obesity (*n* = 68)	*p*
Language of the Intervention Spanish Only or Both Spanish and English	135 (66.50%)	70 (67.31%)	65 (65.66%)	0.80	44 (65.67%)	47 (69.12%)	0.67
Language of Intervention Materials Both Spanish and English	141 (69.46%)	71 (68.27%)	70 (70.71%)	0.71	43 (64.18%)	49 (72.06%)	0.33
Importance of Intervention Leader to be Bilingual ^a^	180 (88.67%)	94 (90.38%)	86 (86.87%)	0.43	60 (89.55%)	59 (86.76%)	0.62
Importance of Intervention Leader to be Hispanic or Latino ^a^	134 (66.01%)	74 (71.15%)	60 (60.61%)	0.11	47 (70.15%)	43 (63.24%)	0.39
Frequency of Meetings ^b^ Willing to attend an intervention that met twice per week	170 (83.74%)	87 (83.65%)	83 (83.84%)	0.97	54 (80.60%)	58 (85.29%)	0.47
Length of Intervention ^b^ Willing to attend an intervention that lasted for about one and a half to two hours on the first day and one and a half hours on the second day	151 (74.38%)	78 (75.00%)	73 (73.74%)	0.84	51 (76.12%)	51 (75.00%)	0.88

Note. ^a^ Moderately, Very, or Extremely Important. ^b^ % Yes. PR = Puerto Ricans; MX = Mexicans.

**Table 2:** Preferences for the Location of the Intervention and Exercise Preferences among All Participants and among Participants with Overweight or Obesity.

Preferences: *n* (%)	Overall (*n* = 203)	All Puerto Ricans (*n* = 104)	All Mexicans (*n* = 99)	*p*	PR with Overweight/ Obesity (*n* = 67)	MX with Overweight/ Obesity (*n* = 68)	*p*
**Location Preferences: *n* (%) ^a^**							
Humboldt Park	153 (75.37%)	94 (90.38%)	59 (59.60%)	<0.0001	61 (91.04%)	40 (58.82%)	<0.0001
University of Illinois at Chicago area	147 (72.41%)	67 (64.42%)	80 (80.81%)	0.009	39 (58.21%)	55 (80.88%)	0.004
Pilsen	98 (48.28%)	35 (33.65%)	63 (63.64%)	<0.0001	23 (34.33%)	46 (67.65%)	0.0001
Little Village	79 (38.92%)	29 (27.88%)	50 (50.51%)	0.003	17 (25.37%)	37 (54.41%)	0.0006
**Exercise Preferences: *n* (%) ^a^**							
How Interested to attend an Exercise Program if the exercise consisted of aerobics with Latin or Salsa Movements	152 (74.88%)	84 (80.77%)	68 (68.69%)	0.047	57 (85.07%)	45 (66.18%)	0.011
How Interested to attend an Exercise Program if the exercise consisted of aerobics with Latin or Salsa Music	143 (70.44%)	78 (75.00%)	65 (65.66%)	0.14	53 (79.10%)	43 (63.24%)	0.042

Note. ^a^ Moderately, Very, or Extremely Interested. PR = Puerto Ricans; MX = Mexicans.

**Table 3:** Preferences for the Location of Intervention and Exercise among All Participants and Participants with Overweight/Obesity.

Cultural Elements: *n* (%)	Overall (*n* = 203)	All Puerto Ricans (*n* = 104)	All Mexicans (*n* = 99)	*p*	PR with Overweight/ Obesity (*n* = 67)	MX with Overweight/ Obesity (*n* = 68)	*p*
Heard of Dichos ^a^	163 (80.30%)	78 (75.00%)	85 (85.86%)	0.052	52 (77.61%)	56 (82.35%)	0.49
Dichos Related to Food or Eating ^a^	97 (59.51%)	44 (56.41%)	53 (62.35%)	0.44	31 (59.62%)	35 (62.50%)	0.76
Dichos Related to Exercise, Physical Activity, or Dancing ^a^	76 (46.63%)	41 (52.56%)	35 (41.18%)	0.15	30 (57.69%)	21 (37.50%)	0.04
Dichos Related to your Body ^a^	89 (54.60%)	43 (55.13%)	46 (54.12%)	0.90	29 (55.77%)	31 (55.36%)	0.97
Dichos Related to Health ^a^	92 (56.44%)	45 (57.69%)	47 (55.29%)	0.76	32 (61.54%)	30 (53.57%)	0.40
How Interested in Participating in an Intervention if it included Dichos(sayings proverbs) in order to make it more culturally meaningful for Latinos ^b^	132 (65.02%)	62 (59.62%)	70 (70.71%)	0.10	37 (55.22%)	46 (67.65%)	0.12
How Interested in Participating in an Intervention if it included Cuentos (stories or storytelling), in order to make it more culturally meaningful for Latinos ^b^	141 (69.46%)	64 (61.54%)	77 (77.78%)	0.012	39 (58.21%)	53 (77.94%)	0.014

Note. ^a^ % Yes. ^b^ Moderately, Very, or Extremely Interested. PR = Puerto Ricans; MX = Mexicans.

## Data Availability

The data that support the findings of this study are available from the corresponding author upon reasonable request.
